# Schoolteachers’ experiences of implementing school-based vaccination programs against human papillomavirus in a Chinese community: a qualitative study

**DOI:** 10.1186/s12889-019-7878-7

**Published:** 2019-11-12

**Authors:** Judy Yuen-man Siu, Albert Lee, Paul K. S. Chan

**Affiliations:** 1 GH339, Department of Applied Social Sciences, Faculty of Health and Social Sciences, The Hong Kong Polytechnic University, Hung Hom, Hong Kong; 2 Jockey Club School of Public Health and Primary Care, Faculty of Medicine, The Chinese University of Hong Kong, Sha Tin, Hong Kong; 3 Department of Microbiology, Faculty of Medicine, The Chinese University of Hong Kong, Sha Tin, Hong Kong

**Keywords:** Papillomavirus vaccine, Perceptions, Barriers, School-based vaccination, Schoolteachers

## Abstract

**Background:**

Cervical cancer was the fourth most common cancer among women worldwide in 2012 and was the eighth most common cancer in 2014 and the eighth greatest cause of female cancer deaths in Hong Kong in 2015. Human papillomavirus (HPV) vaccination has been clinically documented to have a high efficacy in reducing HPV-related cervical intraepithelial neoplasia incidence. Therefore, receiving vaccination is a crucial public health measure to reduce disease burden. Significant others, such as schools and schoolteachers, have prominent influence in shaping adolescents’ health perceptions and behavior. Therefore, the perspective of schools and schoolteachers regarding vaccination can significantly influence students’ acceptance and accessibility of the vaccine. However, few studies have analyzed the perceptions of schoolteachers toward HPV vaccination, and even fewer have concerned how schoolteachers’ perceptions influence their schools’ motivation in implementing school-based HPV vaccination programs. This study was thus conducted to fill this literature gap.

**Methods:**

With a Chinese community as the field site of this study, a qualitative approach of five focus group interviews was conducted with 35 schoolteachers from five primary and eight secondary schools in Hong Kong between July 2014 and January 2015. Thematic content analysis was used for data analysis.

**Results:**

Perceptual, institutional, student and parental, and collaborator barriers interacted to discourage the sampled schoolteachers from organizing school-based HPV vaccination programs. Lack of knowledge regarding HPV vaccination, perception of HPV vaccination as inappropriate given the students’ age, violation of traditional cultural values, lack of perceived needs and perceived risk, opposition from schools, low priority of HPV vaccination over other health education topics, lack of government support, lack of interest from parents and students, and lack of confidence in implementing organizations, all were the mentioned barriers.

**Conclusions:**

The sampled schoolteachers were demotivated to organize school-based HPV vaccination programs because of their perceptions and various social and cultural factors. As significant influencers of adolescent students, schoolteachers and schools should receive more support and information on organizing school-based HPV vaccination programs in the future.

## Background

Cervical cancer was the fourth most common cancer among women worldwide in 2012 [1]. It was also the eighth most common cancer and the ninth greatest cause of female cancer deaths in Hong Kong in 2014 [2]. Infection with high-risk type of human papillomavirus (HPV) can lead to cervical cancer and cancers of the vulva, vagina, penis, anus, and oropharynx, and low-risk types can lead to genital warts [3]. Clinical evidence demonstrates that HPV-associated cancers can be prevented with HPV vaccination [3]. In Hong Kong, the 4- and 9-valent HPV vaccines are suggested for male and female individuals aged 9 years or older [4]. However, young women aged 18 years or younger and women who are not yet sexually active are believed to benefit from the vaccine with highest efficacy [5]. Therefore, school-aged children and young individuals are frequently advised to receive the HPV vaccination [6]. Children are individuals aged 6–12 years and adolescents are people aged 13–18 years.

In Hong Kong, as at the time of writing, the HPV vaccine is an optional vaccine and not included in the Department of Health’s mandatory Hong Kong Childhood Immunization Programme [7]. A low HPV vaccination uptake rate occurred in 2008, with only 768 women receiving the HPV vaccine [8]. This rate did not improve considerably by 2010, and the uptake rate of the HPV vaccination was 7% according to a study among secondary school female students [9]. The Society of Physicians of Hong Kong and The Family Planning Association of Hong Kong have been providing subsidized HPV vaccines under the Youth HPV Prevention Programme to primary and secondary school students since 2011 at a discounted rate of HK$2000 (~US$256) for the whole course [10]. Only in 2016 did the role of the Hong Kong Government in HPV vaccination intensify, with the Community Care Fund under the Hong Kong Government launching an initiative known as the Free Cervical Cancer Vaccination Pilot Scheme, which offers free HPV vaccination to girls aged 9–18 years from low-income families receiving Comprehensive Social Service Assistance [11].

HPV may infect 75% of sexually active people [12]. Although receiving HPV vaccination is an effective method of preventing infection, receiving vaccination is not necessarily a straightforward decision for people to make [13, 14], and many social and cultural factors interact to influence people’s approval of the vaccine.

The health perceptions and behavior of children and teenagers are notably influenced by significant others [15]. Schoolteachers in particular serve as a key medium and role model for imparting knowledge and socializing children and teenagers regarding health knowledge. Schools are the most popular venues for implementing health education and vaccination programs to students in Hong Kong. The home-school-doctor model initiated by The Chinese University of Hong Kong, for instance, features collaboration among schoolteachers, parents, and doctors in enhancing the health of students. This model has employed support from schools and improved the vaccine uptake rate of adolescents to over 30% [16]. The availability of this model was reported to be the most significant independent factor for adolescents’ uptake of the vaccine (OR 26.6; 95% CI 16.4, 41.9) [16]. Therefore, the perspective of schools and schoolteachers can significantly influence the acceptability and accessibility of the vaccine by students and their parents, and thus influence the vaccination rate. Studies have reported on the low acceptability of the HPV vaccine by mothers and female university students in Hong Kong [17, 18]. Although schoolteachers have been observed to play an extraordinary role in modeling student health perceptions and behavior [19], few studies have been conducted toward understanding the attitudes of schoolteachers themselves toward the HPV vaccination, and even fewer concerning how schoolteachers’ perceptions influence their schools’ motivation and decisions to implement school-based HPV vaccination programs. The few studies that have discussed this subject have been conducted in non-Asian countries [20–22] and with a paucity of Chinese-based research.

Many adolescents are oblivious to the importance of receiving HPV vaccination as a preventive health measure [23], partly because of a lack of support and education from schools. Therefore, increasing the awareness of schools and schoolteachers is crucial to motivating adolescents to receive vaccination, thus reducing the prevalence and disease burden of cervical cancer and HPV-associated diseases in Hong Kong. This study investigated how schoolteachers in primary and secondary school students in Hong Kong perceive HPV and HPV vaccines, their experiences regarding implementation of HPV vaccination programs in schools, their thoughts regarding students’ need for receiving the vaccination, and the incentives and barriers for them to implement HPV vaccination programs in their schools.

## Methods

### Data collection

A qualitative approach involving focus group interviews was adopted. The data produced from focus group interviews become saturated by the fifth or sixth session according to the literature [24]. However, the number of focus group interviews conducted in this study was based on whether the data became saturated. Data became saturated in the fourth focus group interview, and five focus group interviews were conducted with 35 schoolteachers—32 female and 3 male teachers—from five primary schools and eight secondary schools. Each focus group contained five to eight participants. Sampled schoolteachers were assigned to the same groups according to school type to ensure they shared common backgrounds, which facilitated discussion and interaction [24].

The schoolteachers were approached by telephone and recruited through purposive sampling from the pool of the Healthy School Project organized by the second and third authors’ institution with the following sampling criteria: (a) aged 20 years or older, (b) worked as a teacher for 5 years or longer, (c) was involved in the design of the health education curriculum and activities for their schools, (d) did not receive medical or health science training, and (e) was Hong Kong Chinese by ethnicity.

An interview question guide (see Additional file 1) was developed on the basis of the past literature regarding HPV vaccination in school settings [7, 20–22] and in Hong Kong [9, 16–18, 25], which enabled the interviews to focus on the research questions. The interview question guide was pilot-tested with those who shared similar characteristics according to the sampling inclusion criteria to ensure that the questions were comprehensible to the participants.

The focus group interviews were conducted between July 2014 and January 2015, with each interview lasting for 1.5–2 h. The first author, who has a background in anthropology and public health, was the facilitator for all interviews to ensure consistency. A research assistant was the observer and note-taker for all interviews and was responsible for taking field notes and observational data during the interviews. The first author prepared interview notes, recording the key themes and observations after each interview. The interviews were conducted in a private room at the first author’s institution. With the consent of the participants, the interviews were audiorecorded. All interviews were conducted in the participants’ mother tongue, Cantonese Chinese, to facilitate free and active discussion. No participant dropped out from the interviews. Each participant received a HK$100 bookshop coupon as an acknowledgment on completion of the interviews.

### Ethical considerations

The study obtained research ethics approval from the Committee on the Use of Human and Animal Subjects in Teaching and Research of Hong Kong Baptist University. Before the interviews, each participant was given a study information sheet outlining the nature of the study and a consent form. The first author clarified enquiries by the participants. Written consent was obtained from the participants for their participation and the academic publication of data. All participants were presented a code to protect their privacy.

### Data analysis

Quick data analysis, which involved checking the questions from the interview question guide constantly and making quick assessments of the interview flow for further probing, was conducted simultaneously with the interviews [26]. Interviews were transcribed verbatim, and two participants from each focus group were asked to check the interview transcripts for accuracy to preclude distortion.

Thematic content analysis was conducted; data saturation, defined as no new themes emerging from the data [24], was achieved. The first author, with a background in anthropology and public health, coded the transcribed interviews. After data cleaning, the raw text of the interviews was read thoroughly for familiarization with the content and then was reread to detect reoccurring themes [27]. Interview transcriptions were analyzed line by line through an inductive coding process [24]. Interview transcriptions were segmented into smaller meaning units [27]. The segments were labeled and then collapsed into categories [27]. The upper level categories were identified based on the research questions, and in vivo coding was conducted [27]. Recurrent categories were highlighted. The overlapping codes and categories were reduced and grouped together to form larger themes with repeated examination and comparison [27]. The codes, categories, and themes deriving from the data, with supporting interview quotes, were documented in a coding table [28]. Observational data was recorded in another codebook, enabling cross-referencing with the interview findings. To enhance data trustworthiness and reliability, the first author conducted a second coding procedure after 2 months from the first coding. The data collection and data analysis procedures of this study conformed to the guidelines of the Consolidated Criteria for Reporting Qualitative Studies (see Additional file 2: Table S1) [29].

## Results

### Participants

All participants, 3 male and 32 female schoolteachers aged 31–50 years, had completed tertiary education; of them, 22 and 13 were from secondary and primary schools, respectively. They had 6–25 years of teaching experience. Only five of them had received the HPV vaccination, and only one had had her daughter vaccinated. None of the children of those who had not been vaccinated had received the immunization, as had none of those teachers’ spouses.

### Barriers to implementing HPV vaccination programs in schools

Perceptual, cultural, institutional, parental, and collaborator barriers were the identified obstacles preventing the sampled schoolteachers from organizing HPV vaccination programs in their schools.

#### Perceptual barriers

##### Lack of knowledge regarding HPV vaccination

Most participants lacked knowledge regarding the HPV vaccine and were uninformed of the efficacy of the vaccine and who should receive it. This prevented them from implementing HPV vaccination programs at the school level:

*Messages regarding the vaccine are unclear. Advertisements just mention that you should get vaccinated as early as possible. However, how early should someone get vaccinated? How effective is the vaccine? Are there any data that can tell us about the efficacy of the vaccine in preventing cervical cancer? There is no information for us. It is difficult to obtain any information about the vaccine from the government. Without any clear information, it is hard for us to encourage [having] the vaccine at school.* (NoVxPri_D)

The nonscientific educational background of several schoolteachers limited their ability to access information concerning the HPV vaccine, which led to difficulties in implementing HPV vaccination programs in their schools:

*It is difficult for us to explain the vaccine to students. For example, it is easy for us to educate students about how to prevent colorectal cancer, but it is hard for us to teach students about preventing cervical cancer. I mean that the concept is difficult for us. You can simply tell students to eat more vegetables and fruits and eat less meat to prevent colorectal cancer. However, how is cervical cancer prevented? You have to see a doctor and do some examinations to diagnose cervical cancer, hence the concept is too difficult for us to tell to students. They do not even know what Pap smears are. The students cannot conceive of the concept of cervical cancer, so it is difficult for us to teach them about the importance of having the vaccine.* (NoVxSec_A)

##### HPV vaccination as irrelevant to their students

Perceiving their students as too young to receive the HPV vaccination was common among participants, particularly among schoolteachers in primary schools:

*Our students are too young to consider receiving the cervical cancer vaccine. They are still primary school students and have not yet reached puberty, so it is too early for them to have this vaccine. They have no idea about cervical cancer and do not even know what a uterus is. I do not think that having the cervical cancer vaccine is an urgent matter for them. When they study in secondary schools, then it will probably be the appropriate time for them to consider having this vaccine.* (NoVxPri_D)

However, even those teaching in secondary schools considered their students as too young to receive the HPV vaccine. They believed that their students lacked knowledge regarding reproductive organs and cervical cancer, which could render the concept of receiving HPV vaccination difficult to impart to their students:

*Even for sixth-former [grade 12] students, it is too early for them to be aware of the presence of the uterus and the risk of cervical cancer. It is too early for them to receive Pap smears and too early for them to have gynecological check-ups. They do not know what a uterus and cervical cancer are. Therefore, how can you educate them to receive the cervical cancer vaccine to prevent against cervical cancer? It is too early for them to understand this concept*. (NoVxSec_A)

The participants often perceived their students as irrelevant to cervical cancer:

*It is a lot easier for students to understand colorectal cancer, I think. Many students have already grasped the concept of colorectal cancer, and thus, it is a lot easier for us to educate them on how to prevent it. Students also show much interest when they hear about endoscopies and colorectal cancer screening. However, cervical cancer is too far away from the students. They are still too young to get this cancer. Therefore, it is very difficult for us to educate them about receiving the cervical cancer vaccine*. (NoVxSec_C)

##### Lack of perceived needs and perceived risk

More than half of the participants perceived that their students did not need to be vaccinated because they believed their students were abstinent:

*In the public health course, some sixth-former [grade 12] students mentioned that cervical cancer is caused by sex but as they had never been dating before nor had any sexual experience, thus they did not regard themselves as being at risk for cervical cancer. They raised an interesting question: Is the cervical cancer vaccination still necessary for them? I agree with the students as well. Furthermore, if sixth-former students do not think that they need to have the vaccine, then the younger students have an even lower need. That is why we have never thought of organizing a cervical cancer vaccination program in our school*. (NoVxSec_A)

Compared with other optional vaccines, such as the seasonal influenza vaccine, the HPV vaccination was often of the lowest priority because the immediate impact of not receiving it was negligible for students and schools:

*Compared with cervical cancer, of course the impact of influenza on students and schools is far greater. When too many students get infected with influenza, we have to report it to the Education Bureau, and class suspension will be compulsory. This will affect students’ learning and our teaching schedule. We may need to use the summer vacation to make up on missed lessons. Therefore, the impact of influenza on students, teachers, and the school operation is far more immediate. However, it is quite difficult to get cervical cancer in schools. I have never heard of a school having a cervical cancer outbreak before. Hence, we prefer having influenza vaccinations rather than cervical cancer vaccinations in schools.* (NoVxSec_A)

Unclear information regarding the length of vaccine efficacy and doubts concerning vaccine safety also affected participants’ sense of perceived needs for their students to receive the vaccination. They consequently preferred waiting for a newer and better HPV vaccine:

*I do not know how long the efficacy lasts after having the cervical cancer vaccine. Is it lifelong or just for several years? If it is only effective for 10 years, then the protection will have been lost when the students get married, and the vaccine will have been wasted. In this case, do the students need to get vaccinated at this early stage? Also, there may be a newer and better vaccine after several years, so there is no need to rush the students for vaccination so early on.* (VxPri_C)

#### Cultural barriers

##### Violation of traditional cultural values

More than half of the participants were concerned that organizing an HPV vaccination program in schools could convey a negative message to students on sex attitudes and worried whether students would misinterpret it to mean that premarital sex was acceptable:

*The students may think that they can do whatever they like and behave irresponsibly in sex after having the cervical cancer vaccine. They may engage in casual sex more easily because they feel safe and think that they no longer need to worry about getting sick after the vaccination. As teachers, we do not want the students to have any misunderstandings; after all, we do not encourage premarital sex*. (NoVxSec_B)

The idea that the HPV vaccination challenged the traditional cultural value of chastity was common among participants. The danger of the disease—here, cervical cancer—was commonly adopted by participants as a fear appeal in an attempt to regulate their students’ sexual attitudes:

*The cervical cancer virus [HPV] is transmitted through sex, and the cervical cancer vaccine is used to prevent this virus. Then, I will worry: If adolescents are vaccinated with the cervical cancer vaccine, will it affect their attitudes toward sex? They may think that they will be free of risk after getting vaccinated, so they may become promiscuous. They may believe that the vaccine can prevent all sex diseases [sexually transmitted diseases], just like many people think that condoms do. Without getting vaccinated, they may be more well-behaved because everyone is afraid of getting sick, not to mention getting cancer. However, once they are vaccinated, they may become more open toward sex.* (NoVxPri_C)

##### Violation of schools’ religious beliefs

The HPV vaccine was perceived as a potential violation of religious beliefs in Catholic schools:

*When my colleagues and I were planning to organize another cervical cancer vaccination program this year, the principal rejected the scheme because of some objections from the [Catholic] Church and the nuns. The nuns were already unhappy that we had organized the vaccination program last year. The Church worried that this vaccine could challenge the value of chastity and encourage promiscuity among students. The opposition from the nuns caused the principal to reject the vaccination program*. (VxSec_G)

#### Institutional barriers

##### Opposition from schools

The perception from school management was also a significant barrier preventing the implementation of the HPV vaccination program in schools. As experienced by the participants, the school management commonly worried about the potential risks of in-school HPV vaccination programs:

*We did not organize the vaccination in school because we worried about the potential negative response to the vaccine suffered by students—I mean the physical response. If they feel unwell after getting vaccinated, what should we do? After all, we are responsible and accountable because the school recommends the vaccination. However, we can never know how the students respond to the vaccine, and this makes us the most worried.* (NoVxSec_B)

Worries of being blamed by parents for postvaccination adverse events were a significant barrier for the participants:

*If the students feel unwell after the vaccination or if they experience more serious side-effects, then the school and schoolteachers have to bear the responsibility. The parents will blame us, and the school will be accountable. You know, most vaccinations in schools are done by nurses, not by doctors. Also, the vaccination venue is in a school, not in a clinic. This really made us worried about liability, so the management staff of my school hesitated to recommend vaccination.* (NoVxSec_A)

##### Low priority of HPV vaccination compared with other health education topics

Compared with other health education topics, HPV vaccination is often designated as a low-priority health education activity in participants’ experience because of the perceived lack of impact on students’ health:

*We have to plan many health education activities in a year. Healthy eating, weight management, and obesity are much more highly prioritized topics, and we do not have time to do more. Whatever you say about vaccinations, I think the influenza vaccination occupies a much higher priority since the needs are more urgent.* (NoVxSec_A)

Parental perception also influenced the participants in prioritizing the health education activities for students:

*If the infection rate of cervical cancer were to keep increasing quickly, then we would give it a higher priority. However, most people think that this vaccine is unimportant, especially at the primary school level. Even parents think that the cervical cancer vaccine is superfluous for their children. Therefore, we would not put the cervical cancer vaccine on our priority list. After all, we have to consider what the parents think.* (NoVxPri_B)

The government education authorities also played a significant role in influencing the planning of health education activities:

*Although we have a degree of flexibility in planning health education activities for our students, we still have to meet the expectations and guidelines of the Education Bureau. It is a must for us to work on drug abuse prevention for students. Other topics such as love, violence, and sex education also are top priorities. If we do not organize those activities, then there are issues with the Education Bureau. After arranging these activities, the timetable for the year’s health education plan is totally full. How can we have time to work on cervical cancer vaccinations?* (NoVxSec_A)

##### Lack of government support

The lack of government support for the HPV vaccine also caused it to be unimportant to the participants, lowering their sense of need for students to receive the vaccination:

*I am not sure if it [HPV vaccine] is really needed. To students, I think the chickenpox vaccine may be more urgently required since it [chickenpox] is highly infectious in the school environment. Also, if the cervical cancer vaccine is really useful for students, then I think the government should already be providing it*. *Subsidies for the vaccine could help. However, the government does not have any standpoint on this vaccine. It appears the vaccine is not important.* [VxPri_C]

This lack of support from the government made it difficult for the participants to organize school-based HPV vaccination programs:

*It seems that the government has never indicated how they would help those who want to have the cervical cancer vaccine. It only subsidizes the influenza vaccine. Without government support, however, the parents have to pay the full fee, which is too expensive for most parents at my school.* (NoVxSec_E)

#### Parental barriers

##### Lack of interest from parents

Parental attitudes also influenced participants’ motivation in organizing HPV vaccination program in schools. Their lack of awareness in having their children vaccinated served to demotivate the participants:

*We have to consider how the parents will respond. The students cannot decide [whether to receive the HPV vaccination], but their parents can. Therefore, you have to deal with the parents first. Most parents regard the cervical cancer vaccine as unimportant for their children. Also, all the vaccines provided in schools for students are free of charge, so if they have to pay, the parents will be mistrustful*. (NoVxSec_D)

Another obstacle was the high price of the HPV vaccine:

*The vaccine costs more than a thousand dollars, which scares off many parents. Most parents are not expected to pay such a high fee in school. They would rather use the money for their children to join a study tour or for hobbies and study classes.* (NoVxSec_A)

#### Collaborator barriers

##### Lack of confidence in the collaborating organizations

The credibility of organizations promoting HPV vaccination affected the motivation and confidence of participants in organizing HPV vaccination programs in their schools. In many cases, they were suspicious of commercial medical companies:

*We often receive pamphlets about the cervical cancer vaccine from pharmaceutical companies and some unknown medical centers. I am skeptical about these organizations because most of them are commercial companies. I think they may just be concerned about making more money through selling more vaccines.* (NoVxSec_A)

Collaboration with organizations perceived as trustworthy could enhance the incentive of organizing HPV vaccination programs in their schools:

*It really depends a lot on the organization. The Cancer Crusade Angel [The Cancer Crusade Angels Services Society of Hong Kong] has been very keen on promoting the cervical cancer vaccine at our school. The nurses there are very warm-hearted and sincere, and they called our school many times about the vaccine. We trust this organization a lot and have been collaborating with them for years. If the Cancer Crusade Angel does not arrange the vaccination, then our school will stop offering it.* (VxSec_E)

Universities were another trustworthy collaborator for most participants:

*We have greater confidence when a university helps our school to organize cervical cancer vaccinations for students. Not only the school, but also the parents feel a lot more confident.* (VxSec_I)

## Discussion

Schoolteachers’ perceptions of and beliefs about the HPV vaccine can have a decisive role in influencing students’ accessibility to and acceptance of the vaccine. School-based interventions can positively influence beliefs regarding the prevention of HPV and increase HPV vaccination rates in adolescents [30]. However, as the findings indicate, organizing a school-based HPV vaccination program is never straightforward. Perceptual, cultural, institutional, parental, and collaborator barriers interact to discourage the sampled schoolteachers from organizing vaccination programs.

HPV vaccine perceptions among the sampled schoolteachers played a crucial role in motivating or discouraging them from organizing school-based HPV vaccination programs. The participants commonly lacked knowledge regarding the HPV vaccine, particularly those from nonorganizing schools. They felt unclear regarding use of the vaccine, vaccine efficacy, and the target population. This uncertainty led to their hesitation in organizing school-based vaccination programs. However, participants who had organized HPV vaccination programs in their schools had more knowledge of HPV and the HPV vaccine, and a close relationship between personal vaccination status of participants and a tendency to organize school-based HPV vaccination programs was observed. The five participants who had received the HPV vaccine had organized HPV vaccination programs in their schools. This demonstrated a positive relationship between knowledge of HPV and the HPV vaccine and motivation to organize school-based HPV vaccination programs, which coincided with findings from South Africa [31]. Therefore, providing more public health education regarding HPV and the HPV vaccination to schoolteachers may empower them to implement school-based vaccination programs and related health education for students.

Not believing their students needed to receive the HPV vaccine, the participants were not inspired to organize school-based HPV vaccination programs. They perceived the concept of cervical cancer as too difficult for their students to comprehend because they had not yet reached an age in which they were generally considered vulnerable. Furthermore, the term used to refer to the HPV vaccine—“cervical cancer vaccine”—throughout the interviews explained this attitude. According to the Sapir–Whorf Hypothesis, language affects a person’s thinking. The language and vocabulary used can influence speakers’ perceptions and thus affect their attitudes, behavior, and worldview [32]. Referring to the HPV vaccine as the cervical cancer vaccine reflected the participants’ perceptions that the vaccine is merely for preventing cervical cancer, a disease that was perceived as irrelevant to their students. Although such attitudes were also present among those who had organized school-based HPV vaccination programs, these participants had a more positive view, regarding the vaccination program as a form of health education and brought to their students the message of disease prevention.

Although irrelevance to student needs was a notable barrier preventing school-based HPV vaccination programs, this did not necessarily indicate that the participants would only organize health education activities they thought were relevant to their students’ needs. Health education activities regarding colorectal cancer prevention, for instance, were common in the participants’ schools, even though colorectal cancer rarely affects adolescents according to the medical literature [33]. We argued that a key reason for this difference in the attitude is the traditional cultural values and stereotypes regarding cervical cancer, HPV, and the HPV vaccine in Chinese communities. These and other factors interacted to explain the participants’ and their schools’ reluctance to organize school-based HPV vaccination programs.

Concerns of students’ sexual attitudes were an obstacle. Cervical cancer is caused by sexual activity [34], thus organizing school-based HPV vaccination programs is perceived as demonstrating schools’ approval for premarital sexual behavior, which violates the traditional Chinese cultural value of chastity before marriage. This impedes young adults’ HPV vaccination uptake [17, 18]. Because schools are critical social institutions of socialization, contributing to how students behave in accordance with society’s expected social and cultural norms and values, schools are thus not expected to organize programs that break with these values, including the participants and other stakeholders, such as the school management and parents. Moreover, organizing school-based HPV vaccination programs was even less straightforward for schools with certain religious backgrounds, in which the support of the school management was entirely lacking. An effective health promotion program must address participants’ perception of social norms [35] and enriching health education in this area would cultivate a positive normative belief in the desired practice by inducing the motivation to comply (i.e., vaccine uptake).

All these issues result in HPV vaccination often having the lowest priority in schools’ health education curricula. To the participants and school management, the immediate impact of cervical cancer on students is negligible because of their belief in their students’ abstinence and the moral and cultural implications of the HPV vaccination that prevented the participants from organizing such health-enhancing, but morally and culturally sensitive, programs. Therefore, other health education and vaccination activities that warranted immediate attention and that lacked moral and cultural implications, such as the seasonal influenza vaccination and colorectal cancer education, were much higher on the agenda. The participants preferred to employ the fear appeal of cervical cancer to regulate students’ sexual attitudes and behaviors.

As a powerful social institution, the government health and education authorities played a prevalent role in downplaying the importance of HPV vaccination. The government health authorities did not openly promote the importance of HPV vaccination to the public before the government’s Policy Address of 2018. This in turn influenced the participants’ perceived need of providing vaccination to their students. Under this policy influence, it was extremely difficult for schoolteachers to obtain information, education, and support regarding the HPV vaccine from the authorities, making the HPV vaccine appear unimportant. Therefore, government health authorities and their vaccination policies serve a remarkable role in affecting participants’ perceived (un) importance in vaccinating students. The perceived unimportance of vaccinating students against HPV, however, may change in future, as the government will introduce free HPV vaccination to school girls of particular age-groups starting from the 2019/2020 school year [36]; hopefully this will alter the perceptions of HPV vaccine in the community.

Unlike in the United States, where mothers are willing to vaccinate their daughters against HPV in a school-based format [37], this study’s participants noted a contrasting opinion from parents. Parents’ attitudes affected the participants’ motivation. Parents are the predominant partners of schools and schoolteachers according to the home-school-doctor model [16]. Without the support of parents, the participants could not justify the organization of school-based HPV vaccination programs.

The nature of collaborating organizations also played a key role in motivating participants to organize school-based HPV vaccination programs. The credibility of health care is diminished when associated with commercial companies because of the stereotype of commerce as profit-making, leading to people’s suspicion and lack of confidence in health care measures [38]. Nongovernmental health organizations and universities were observed to be the most trustable and credible institutions according to the participants’ perceptions. Moreover, the involvement of nongovernmental organizations in school-based vaccination programs achieved an 80% acceptance rate among students and parents [39], supporting the adoption of the home-school-doctor model as a meaningful approach to school-based HPV vaccination programs [16]. Therefore, collaborating with nongovernmental health organizations and universities could be a feasible direction for school-based HPV vaccination programs in the future. Furthermore, school-based health centers in the United States were reported to improve HPV vaccine uptake among adolescents because they offer convenience and do not affect school or work [40]. Despite the lack of school-based health centers in primary and secondary schools in Hong Kong, with the support of the literature indicating school-based format can enhance vaccination motivation [37, 40–43] and offer trust to parents [44], the home-school-doctor model could be considered an alternative in the implementation of school-based HPV vaccination programs [16] in the future to improve vaccine uptake.

Consistent with literature [45], the high cost of the vaccine affected the perceived importance of receiving vaccination. Although providing monetary subsidies could have helped increase vaccination incentives, many other social and cultural factors interacted to affect the participants’ perceptions and thus their motivations in organizing school-based HPV vaccination programs. Further public health education regarding HPV and information on the HPV vaccine must be provided to sway these crucial stakeholders. As students only have limited autonomy in making vaccination decisions and are still undergoing socialization from their significant others, such as schoolteachers, providing public health education regarding HPV and the HPV vaccine to schoolteachers is crucial to enhancing their awareness on the importance of receiving vaccination, which can in turn encourage students to adopt this preventive health behavior.

## Limitations

This was a qualitative study, including only 35 schoolteachers as participants, with a significant imbalance between male and female participants. Therefore, it could provide a thematic understanding of the resistance to HPV vaccination programs but could not quantitate the proportion of schoolteachers who have these concerns or objectively measure whether these barriers are critical for the rest of population. Future studies sampling more schoolteachers from more field sites with a balanced gender ratio could add more credibility to investigating the prospect of organizing school-based HPV vaccination programs.

## Conclusion

The promotion of HPV vaccination to adolescents is crucial for reducing the disease burden of cervical cancer and other HPV-associated diseases in the long term. However, adolescents have limited autonomy in making decisions for their preventive health, thus schools are important institutions of socialization, and schoolteachers’ attitudes and beliefs play a decisive role in shaping students’ health perceptions and preventive health behavior. With the interacting social and cultural barriers identified, schoolteachers and schools should receive more support and information regarding HPV vaccines to facilitate the organization of school-based HPV vaccination programs in the future.

## Supplementary information

**Additional file 1.** Interview question guide for focus group interviews

**Additional file 2: Table S1.** Cross-checking with the Consolidated Criteria for Reporting Qualitative Research (COREQ): 32-item checklist

## Data Availability

The datasets generated and analyzed during the current study are not publicly available for participants’ confidentiality but are available from the corresponding author on reasonable request.

## Abbreviations

HPV: Human papillomavirus
