# Supplementary material for: Schoolteachers’ experiences of implementing school-based vaccination programs against human papillomavirus in a Chinese community: a qualitative study
Source: BMC Public Health. 2019 Nov 12;19:1514. doi: 10.1186/s12889-019-7878-7 (PMC6852998; doi:10.1186/s12889-019-7878-7)
Supplement: Supplementary file 1 — Additional file 1. Interview question guide for focus group interviews [file 12889_2019_7878_MOESM1_ESM.docx]

**Appendix 1. Interview question guide for focus group interviews**

Questions:

*For those teachers who have organized HPV vaccination program in schools*

1. Perception and behavior
2. To you, what is HPV?
3. How do you think about the transmission route of HPV?
4. How do you think about the danger of HPV? Why?
5. Have you ever heard about HPV vaccines? If yes, from what sources?
6. Do you know what HPV vaccines are for? / What diseases can HPV vaccines prevent?
7. Have you / your wife and/or daughters received HPV vaccination? Why? (discover about the influence from significant others to students)
8. Implementation in schools
9. How do you think about the needs of your students in receiving HPV vaccination? Do you think they need to receive it? Why? [Probe informants about if they think male students have the needs to receive the vaccine, and why.]
10. How do you think about the side effects / potential harms on students if they receive HPV vaccines?
11. To you, what age can be suitable in receiving HPV vaccination?
12. (Base on 7 & 8) Does your perception match with the school’s? If not, how did your school think on this?
13. Why did your school join the HPV vaccination program / organize the HPV vaccination program for your students? (motivations)
14. What was your / your colleagues / your school’s experience? Can you share some of the experiences with us? (can discover if there were any problems and difficulties in implementation)
15. Do you think the program was running smoothly? Why?
16. What were the difficulties in implementing such a program in your school? What are the sources of these difficulties? (Barriers in a sense. Parents? If Christian schools / schools with religious background, probe more about religion issue.)
17. How did you / your school tackle these difficulties?
18. How did students’ parents think about the HPV vaccination program in your school?
19. How would you evaluate about this program in your school? Do you think it was successful? Why? What have been achieved to this outcome?
20. If you need to organize another HPV vaccination program in your school, what can be done better?
21. Demographic data (form)

- Age

- Sex

- Marital status

- Religious belief (self and school)

- Subjects taught

- Years of experience as a teacher

- HPV vaccination status [self, spouse, daughter(s)]

*For those teachers who did not organize HPV vaccination program in schools*

1. Perception and behavior
2. To you, what is HPV?
3. How do you think about the transmission route of HPV?
4. How do you think about the danger of HPV? Why?
5. Have you ever heard about HPV vaccines? If yes, from what sources?
6. Do you know what HPV vaccines are for? / What diseases can HPV vaccines prevent?
7. Have you / your wife and/or daughter(s) received HPV vaccination? Why? (discover about the influence from significant others to students)
8. Implementation in schools
9. How do you think about the needs of your students in receiving HPV vaccination? Do you think they need to receive it? Why? [Probe informants about if they think male students have the needs to receive the vaccine, and why.]
10. How do you think about the side effects / potential harms on students if they receive HPV vaccines?
11. To you, what age can be suitable in receiving HPV vaccination?
12. (Base on 7 & 8) Does your perception match with the school’s? If not, how does your school think on this?
13. Have you school ever had considered about organizing the HPV vaccination program for your students? If the school has considered, why it is still not yet implemented?
14. What are the barriers in implementing such a program in your school? What are the sources of these barriers? (Parents? If Christian schools / schools with religious background, probe more about religion issue.)
15. What was your / your colleagues / your school’s experience in this considering and decision-making process? Can you share some of the experiences with us? (can discover if there were any problems and difficulties in implementation that prevent the school from joining the program)
16. How did you / your school tackle these barriers?
17. How did students’ parents think about the HPV vaccination program in your school?
18. What can be done to motivate your school in organizing the HPV vaccination for your students?
19. Demographic data (form)

- Age
- Sex
- Marital status
- Religious belief (self and school)
- Subjects taught
- Years of experience as a teacher
- HPV vaccination status [self, spouse, daughter(s)]
