# Supplementary material for: Schoolteachers’ experiences of implementing school-based vaccination programs against human papillomavirus in a Chinese community: a qualitative study
Source: BMC Public Health. 2019 Nov 12;19:1514. doi: 10.1186/s12889-019-7878-7 (PMC6852998; doi:10.1186/s12889-019-7878-7)
Supplement: Supplementary file 2 — Additional file 2: Table S1. Cross-checking with the Consolidated Criteria for Reporting Qualitative Research (COREQ): 32-item checklist [file 12889_2019_7878_MOESM2_ESM.docx]

**Table 1. Cross-checking with the Consolidated Criteria for Reporting Qualitative Research (COREQ): 32-item checklist (Tong et al., 2007)**

| **Domain 1: Research team and reﬂexivity** | |
| --- | --- |
| Personal Characteristics | |
| 1. Interviewer/facilitator: Which author/s conducted the interview or focus group? | JYS (Methods, data collection, paragraph 5) |
| 2. Credentials: What were the researcher’s credentials? E.g. PhD, MD | JYS: Ph.D, M.Phil. B.B.Sc.  AL: MBBS, MD, MPH, FRCP, FRACGP, FFPH, FHKAM (FamMed), FHKCFP, DCH.  PKC: MBBS, MSc, MD, FRCPath, FHKCPath, FHKAM (Pathology).  (NA) |
| 3. Occupation: What was their occupation at the time of the study? | JYS: Assistant Professor.  AL: Professor (Clinical).  PKC: Professor (Clinical).  (Cover page) |
| 4. Gender: Was the researcher male or female? | JYS: Female.  AL: Male.  PKC: Male.  (Cover page) |
| 5. Experience and training: What experience or training did the researcher have? Relationship with participants | JYS: Anthropology, Public Health.  AL: Medicine, Family Medicine.  PKC: Medicine, Pathology.  The three researchers had no relationship with the participants at the time of study.  (Methods, data collection, paragraph 5; data analysis, paragraph 2) |
| 6. Relationship established: Was a relationship established prior to study commencement? | The three researchers had no relationship established with the participants prior the study. However, the schools that the participants were working for had participated in another Healthy School Project conducted by AL. (Methods, data collection, paragraph 3) |
| 7. Participant knowledge of the interviewer: What did the participants know about the researcher? e.g. personal goals, reasons for doing the research | The participants did not know about the interviewer prior the study. (Methods, data collection, paragraph 5; ethics consideration, paragraph 1) |
| 8. Interviewer characteristics: What characteristics were reported about the interviewer/facilitator? e.g. Bias, assumptions, reasons and interests in the research topic | The interviewer has published several journal articles about the perceptions of having HPV vaccination among mothers, university female students, and men. (Methods, data collection, paragraph 5) |
| **Domain 2: Study design and theoretical framework** | |
| 9. Methodological orientation and Theory: What methodological orientation was stated to underpin the study? e.g. grounded theory, discourse analysis, ethnography, phenomenology, content analysis | Thematic content analysis (Methods, data analysis, paragraph 2) |
| 10. Participant selection Sampling: How were participants selected? e.g. purposive, convenience, consecutive, snowball | Purposive sampling (Methods, data collection, paragraph 3) |
| 11. Method of approach: How were participants approached? e.g. face-to-face, telephone, mail, email | Telephone (Methods, data collection, paragraph 3) |
| 12. Sample size: How many participants were in the study? | 5 focus groups with 35 teachers (Methods, data collection, paragraph 2) |
| 13. Non-participation: How many people refused to participate or dropped out? Reasons? Setting | No participants refused to participate or dropped out from the interviews. (Methods, data collection, paragraph 5) |
| 14. Setting of data collection: Where was the data collected? e.g. home, clinic, workplace | The focus group interviews were conducted in a private room of JYS’s institution. (Methods, data collection, paragraph 5) |
| 15. Presence of non-participants: Was anyone else present besides the participants and researchers? | One research assistant served as the observer and note-taker for all the interviews. (Methods, data collection, paragraph 5) |
| 16. Description of sample: What are the important characteristics of the sample? e.g. demographic data, date | (a) aged 25 to 60 years at the time of study,  (b) had been working as a teacher for five years or more,  (c) had been involving in the design of the health education curriculum and activities for their schools,  (d) had not received medical and/or health science training,  (e) able to understand and speak Cantonese Chinese, and  (f) was Hong Kong Chinese by ethnicity  (Methods, data collection, paragraph 3; Results, the participants, paragraph 1) |
| Data collection | |
| 17. Interview guide: Were questions, prompts, guides provided by the authors? Was it pilot tested? | The interview question guide was developed with the continuous discussion and consensus among the three authors. The questions were developed basing on the past literature about HPV vaccination in school setting as well as on the three authors’ studies about HPV vaccination in Hong Kong. The interview question guide was pilot-tested with those who shared the similar characteristics according to the sampling inclusion criteria to ensure the questions were comprehensible to the participants. (Methods, data collection, paragraph 4) |
| 18. Repeat interviews: Were repeat interviews carried out? If yes, how many? | No repeated interviews were conducted. (NA) |
| 19. Audio/visual recording: Did the researcher use audio or visual recording to collect the data? | Audio-recording was used. (Methods, data collection, paragraph 5) |
| 20. Field notes: Were ﬁeld notes made during and/or after the interview or focus group? | A research assistant was responsible for taking field notes and observational data during the interviews. Besides, the first author prepared interview notes, recording the key themes and observation, after each interview. (Methods, data collection, paragraph 5) |
| 21. Duration: What was the duration of the interviews or focus group? | 1.5 to 2 hours for each focus group interview. (Methods, data collection, paragraph 5) |
| 22. Data saturation: Was data saturation discussed? | Yes, data saturation was achieved. (Methods, data analysis, paragraph 2) |
| 23. Transcripts returned: Were transcripts returned to participants for comment and/or correction? | Yes. Two participants of each focus group were asked to check the interview transcripts for accuracy, ensuring no transcribed data was distorted. (Methods, data analysis, paragraph 1) |
| **Domain 3: Analysis and ﬁndings** | |
| 24. Data analysis: Number of data coders How many data coders coded the data? | The first author coded the transcribed interviews. (Methods, data analysis, paragraph 2) |
| 25. Description of the coding tree: Did authors provide a description of the coding tree? | The codes, categories, and themes deriving from the data, with supporting interview quotes, were documented in a coding table. (Methods, data analysis, paragraph 2) |
| 26. Derivation of themes: Were themes identiﬁed in advance or derived from the data? | Inductive coding was used. Themes were derived from data. (Methods, data analysis, paragraph 2) |
| 27. Software: What software, if applicable, was used to manage the data? | No software was used in data analysis. All the analyzed data and coding tables were managed in the code book document in Microsoft Word. (Methods, data analysis, paragraph 2) |
| 28. Participant checking: Did participants provide feedback on the ﬁndings? | Yes. Participant checking was conducted as the participants of each focus group were invited to provide feedbacks on the analyzed data to ensure the findings had no distortion on their original meaning. (Methods, data analysis, paragraph 1) |
| Reporting | |
| 29. Quotations presented: Were participant quotations presented to illustrate the themes / ﬁndings? Was each quotation identiﬁed? e.g. participant number | Yes, participant quotations were presented with informant code. (Results, p.10-p.19) |
| 30. Data and ﬁndings consistent: Was there consistency between the data presented and the ﬁndings? | Yes. (Results, p.10-p.19) |
| 31. Clarity of major themes: Were major themes clearly presented in the ﬁndings? | Yes. (Results, p.10-p.19) |
| 32. Clarity of minor themes: Is there a description of diverse cases or discussion of minor themes? | Yes. (Results, p.10-p.19) |
